# Supplementary material for: Explainable one-class feature extraction by adaptive resonance for anomaly detection in quality assurance
Source: PLoS One. 2025 Jun 10;20(6):e0321968. doi: 10.1371/journal.pone.0321968 (PMC12151409; doi:10.1371/journal.pone.0321968)
Supplement: Attachment — Submitted filename: PLOS_Response_newfile.pdf [file pone.0321968.s002.pdf]

Response to reviewers

Please find enclosed the revised version of our manuscript "Explainable one-class feature extraction by adaptive resonance for anomaly detection in quality assurance" (# PONE-D-24-09697). We thank the editor and reviewers for their valuable comments, which helped improve the quality of our manuscript. We have carefully addressed each comment in the revision as itemized in this letter. For clarity, the comments are in black, our response to each comment is in darkgreen. The changes in the manuscript text are marked with blue, and are tagged by the comment number for reference (e.g., R1C2 for Reviewer 1, Comment 2).

The changes include major revisions in the Introduction, Related Work, Discussion, and Conclusion sections, and some minor changes elsewhere.

Reviewer 1

1. R1C1: Introduction. The structure of the paper should be improved. In particular, the introduction section should be more structured, faster, and cover the key five elements: motivation, objectives, literature gap and paper's contributions to the literature, justification of the methodology, and summary of the results. Furthermore, in the introduction section, the authors clearly describe the added value of their analysis and the positioning in light of previous findings.

   We significantly re-wrote the Introduction section as you suggested and moved most of the previous introduction to a new Related Work section. The Related Work section includes an expanded literature review as requested in comments R1C1, R1C2, R1C4, and R2C3.

2. R1C2: Related Work. I suggest the authors to analyze more papers that are recently published. Moreover, make sure that literature review is linked to a systematic.

   We have updated the literature review with the following additional citations, which include several papers from 2023 and 2024: Commission (2021); Gorokhov et al. (2017); Kehayias et al. (2023); Li et al. (2023); Lundberg and Lee (2017); Ribeiro et al. (2016, 2018); Saab et al. (2024); Salam et al. (2024); Union (2024)

3. R1C3: The method used should be compared with various methods and the results should be strengthened. The methodological explanation of the method used should be made with the help of assumptions and formulas.

   We added a simple explanation of the main idea behind Adaptive Resonance Theory at the beginning of the Methods section. Additionally, we extensively compare the various methods and their results in the Discussion section.

4. R1C4: The results should be discussed more thoroughly in light of the existing literature. This is a very important point to address.

   We included four additional papers: Altman et al. (2015); Jensen et al. (2018); Kalendralis et al. (2023); Kehayias et al. (2023). Our study stands out by using a comprehensive dataset with over 100 variables and real erroneous plans, unlike previous works that focused on specific error types, lower-dimensional data, or simulated errors. Our one-class approach overcomes the limitations of requiring erroneous plans for training, providing a more robust and adaptable framework for detecting a wide range of unacceptable plans in radiation therapy. This discussion is highlighted in blue on page 17 of the revised document.

5. R1C5: In the conclusion section, a more detailed discussion of the policy implications would make the paper richer and more informative for investors and policymakers.

We added explanation on page 20, in accordance with the most prominent AI regulation document, AI Act published by European Union Union (2024).

Reviewer 2

1. R2C1: Provide more examples or case studies to demonstrate the practical implementation of the proposed model in clinical settings. This would help in illustrating its applicability and effectiveness in real-world scenarios.

   We added discussions about implementation and limitations in the Discussion section on page 20. Additional studies have been included in the discussion section (See R1C3 and R1C4 on page 17), comparing their approaches, their results, and some of the implications of the clinical implementation Kalendralis et al. (2023).

2. R2C2: Expand the discussion on the limitations and challenges of your approach, especially focusing on situations where the model may face difficulties due to the unique characteristics of individual treatment plans or variations in equipment across different healthcare facilities.

   We added additional discussion of implementation challenges and limitations in the Discussion section.

3. R2C3: Include a more detailed comparative analysis with other anomaly detection models that have been applied in similar healthcare settings. Highlighting the specific improvements your model offers over these existing methods would provide a clearer context for your contributions.

   The Related Work section now includes a comparison with alternative methods, highlighting that deep learning approaches often require additional algorithms for interpretation, lacking inherent interpretability.

4. R2C4: Discuss the model's adaptability to other types of quality assurance tasks outside of radiotherapy, potentially broadening the scope of your findings.

   We added a paragraph about other possible applications in the Discussion section.

5. R2C5: Enhance the explanation of how the adaptive resonance features work in layman's terms, to make the paper more accessible to readers who are not specialists in machine learning or medical physics.

   See R1C3.

6. R2C6: Consider the ethical implications of automating such critical aspects of healthcare, particularly the reliance on algorithmic decisions in clinical environments. Discuss safeguards that could be implemented to prevent potential negative outcomes.

   See R1C5.

7. R2C7: Propose future directions for this research, especially in terms of integrating this technology with other AI-driven tools used in healthcare, which could lead to a more comprehensive system for automated quality assurance across various treatment modalities.

   We added a paragraph about future directions in the Conclusion section.

8. R2C8: Cite this `https://doi.org/10.1109/ACCESS.2024.3373697` to improve your literature.

   This paper (Salam et al., 2024) was cited as part of R2C4.

References

MB Altman, JA Kavanaugh, HO Wooten, OL Green, TA DeWees, H Gay, WL Thorstad, H Li, and S Mutic. A framework for automated contour quality assurance in radiation therapy including adaptive techniques. *Physics in Medicine & Biology*, 60(13):5199, 2015.

European Commission. Laying down harmonised rules on artificial intelligence (artificial intelligence act) and amending certain union legislative acts. *Eur Comm*, 106:1–108, 2021.

Oleg Gorokhov, Mikhail Petrovskiy, and Igor Mashechkin. Convolutional neural networks for unsupervised anomaly detection in text data. In *International Conference on Intelligent Data Engineering and Automated Learning*, pages 500–507. Springer, 2017.

N Jensen, K Boye, S Damkjær, and I Wahlstedt. Impact of automation in external beam radiation therapy treatment plan quality control on error rates and productivity. *International Journal of Radiation Oncology, Biology, Physics*, 102(3):S149–S150, 2018.

Petros Kalendralis, Samuel MH Luk, Richard Canters, Denis Eyssen, Ana Vaniqui, Cecile Wolfs, Lars Murrer, Wouter van Elmpt, Alan M Kalet, Andre Dekker, et al. Automatic quality assurance of radiotherapy treatment plans using bayesian networks: A multi-institutional study. *Frontiers in oncology*, 13:1099994, 2023.

CE Kehayias, D Bontempi, S Quirk, S Friesen, JS Bredfeldt, MA Huynh, H Aerts, RH Mak, and CV Guthier. Deep learning-based automated quality assurance for palliative spinal treatment planning in radiotherapy. *International Journal of Radiation Oncology, Biology, Physics*, 117(2): S50, 2023.

Zhong Li, Yuxuan Zhu, and Matthijs Van Leeuwen. A survey on explainable anomaly detection. *ACM Transactions on Knowledge Discovery from Data*, 18(1):1–54, 2023.

Scott M Lundberg and Su-In Lee. A unified approach to interpreting model predictions. *Advances in neural information processing systems*, 30, 2017.

Marco Tulio Ribeiro, Sameer Singh, and Carlos Guestrin. " why should i trust you?" explaining the predictions of any classifier. In *Proceedings of the 22nd ACM SIGKDD international conference on knowledge discovery and data mining*, pages 1135–1144, 2016.

Marco Tulio Ribeiro, Sameer Singh, and Carlos Guestrin. Anchors: High-precision model-agnostic explanations. *Proceedings of the AAAI Conference on Artificial Intelligence*, 32(1), 2018.

Khaled Saab, Tao Tu, Wei-Hung Weng, Ryutaro Tanno, David Stutz, Ellery Wulczyn, Fan Zhang, Tim Strother, Chunjong Park, Elahe Vedadi, Juanma Zambrano Chaves, Szu-Yeu Hu, Mike Schaekermann, Aishwarya Kamath, Yong Cheng, David G. T. Barrett, Cathy Cheung, Basil Mustafa, Anil Palepu, Daniel McDuff, Le Hou, Tomer Golany, Luyang Liu, Jean baptiste Alayrac, Neil Houlsby, Nenad Tomasev, Jan Freyberg, Charles Lau, Jonas Kemp, Jeremy Lai, Shekoofeh Azizi, Kimberly Kanada, SiWai Man, Kavita Kulkarni, Ruoxi Sun, Siamak Shakeri, Luheng He, Ben Caine, Albert Webson, Natasha Latysheva, Melvin Johnson, Philip Mansfield, Jian Lu, Ehud Rivlin, Jesper Anderson, Bradley Green, Renee Wong, Jonathan Krause, Jonathon Shlens, Ewa Dominowska, S. M. Ali Eslami, Katherine Chou, Claire Cui, Oriol Vinyals, Koray Kavukcuoglu, James Manyika, Jeff Dean, Demis Hassabis, Yossi Matias, Dale Webster, Joelle Barral, Greg Corrado, Christopher Semturs, S. Sara Mahdavi, Juraj Gottweis, Alan Karthikesalingam, and Vivek Natarajan. Capabilities of gemini models in medicine, 2024.

Abdu Salam, Mohammad Abrar, Farhan Amin, Faizan Ullah, Izaz Ahmad Khan, Bader Fahad Alkhamees, and Hussain AlSalman. Securing smart manufacturing by integrating anomaly detection with zero-knowledge proofs. *IEEE Access*, 12:36346–36360, 2024. doi: 10.1109/ACCESS.2024.3373697.

European Union. Artificial intelligence act. european parliamentary research service, 2023, 2024.
