# Supplementary material for: Explainable one-class feature extraction by adaptive resonance for anomaly detection in quality assurance
Source: PLoS One. 2025 Jun 10;20(6):e0321968. doi: 10.1371/journal.pone.0321968 (PMC12151409; doi:10.1371/journal.pone.0321968)
Supplement: Attachment — Submitted filename: PLOS_Response_Feb25.pdf [file pone.0321968.s003.pdf]

Response to reviewers

We again thank the reviewers for their thoughtful comments to improve our manuscript, "Explainable one-class feature extraction by adaptive resonance for anomaly detection in quality assurance" (# PONE-D-24-09697). We have revised the manuscript based on this feedback, and itemized each comment and our response below. Changes in the manuscript are marked in blue and tagged with the corresponding comment number (e.g., R2C1 for Reviewer 2, Comment 1).

Reviewer 2

1. The paper needs to check for spelling, typo and grammatical mistakes. The rest of the paper is now ok.

   We have carefully re-read the manuscript, and corrected typos and made some small improvements for style and consistency. For ease of reading, we did not annotate these changes in the manuscript.

Reviewer 3

1. **R3C1** Although additional citations were included, the review could benefit from a systematic and thematic organization. There are still areas where the links between referenced studies and the proposed method remain unclear. The inclusion of comparative analysis with alternative methods is appreciated, but the discussion is surface-level. A deeper dive into why the proposed method outperforms others with specific examples would be more impactful.

   We have revised multiple parts of the manuscript to improve the connection between previous studies and our proposed method:

   - We clarified why handling sparsity in high-dimensional feature spaces is a key challenge in RT planning and explicitly referenced prior work addressing this issue.
   - We strengthened the explanation of why binary classification methods are insufficient for RT QA, emphasizing the risk of failing to detect novel anomalies.
   - We improved the description of how our OART method incorporates feature selection to enhance anomaly detection while maintaining interpretability.

2. **R3C2** Figures and Tables - Ensure clarity and alignment with the narrative.

   We have carefully reviewed all figures and tables in the manuscript to ensure their clarity and alignment with the surrounding narrative. If there are any additional specific issues with the tables and figures, please let us know so we can make the appropriate revisions.
